# Out of Africa: A Molecular Perspective on the Introduction of Yellow Fever Virus into the Americas

**DOI:** 10.1371/journal.ppat.0030075

**Published:** 2007-05-18

**Authors:** Juliet E Bryant, Edward C Holmes, Alan D. T Barrett

**Affiliations:** 1 Department of Pathology, University of Texas Medical Branch, Galveston, Texas, United States of America; 2 Center for Biodefense and Emerging Infectious Diseases, University of Texas Medical Branch, Galveston, Texas, United States of America; 3 Institute for Human Infections and Immunity, University of Texas Medical Branch, Galveston, Texas, United States of America; 4 Institut Pasteur, National Center for Laboratory and Epidemiology, Vientiane, Lao People's Democratic Republic; 5 Department of Biology, The Pennsylvania State University, University Park, Pennsylvania, United States of America; 6 Center for Infectious Disease Dynamics, The Pennsylvania State University, University Park, Pennsylvania, United States of America; 7 Fogarty International Center, National Institutes of Health, Bethesda, Maryland, United States of America; National Institutes of Health, United States of America

## Abstract

Yellow fever virus (YFV) remains the cause of severe morbidity and mortality in South America and Africa. To determine the evolutionary history of this important reemerging pathogen, we performed a phylogenetic analysis of the largest YFV data set compiled to date, representing the *prM/E* gene region from 133 viral isolates sampled from 22 countries over a period of 76 years. We estimate that the currently circulating strains of YFV arose in Africa within the last 1,500 years and emerged in the Americas following the slave trade approximately 300–400 years ago. These viruses then spread westwards across the continent and persist there to this day in the jungles of South America. We therefore illustrate how gene sequence data can be used to test hypotheses of viral dispersal and demographics, and document the role of human migration in the spread of infectious disease.

## Author Summary

Throughout the 18th and 19th centuries, yellow fever was one of the most dreaded of diseases in New and Old world port cities. Large-scale epidemics of yellow fever helped shape colonial expansion in both the Americas and in Africa, and the medical and scientific developments associated with control of the virus have been a favored topic of historians for many years. The most commonly cited hypothesis of the origin of yellow fever virus (YFV) in the Americas is that the virus was introduced from Africa, along with *Aedes Aegypti* mosquitoes, in the bilges of sailing vessels during the slave trade. Although the hypothesis of a slave trade introduction is often repeated, it has not been subject to rigorous examination using gene sequence data and modern phylogenetic techniques for estimating divergence times. Herein we have assembled a comprehensive data set of gene sequences for YFV, which we used to infer the time-scale and evolutionary history of YFV. These data show that the spread of YFV to the Americas corresponds closely with the routes and timing of the slave trade. Overall, this study demonstrates how molecular epidemiological studies can provide new insight into debates on the origin and spread of infectious disease.

## Introduction

Few diseases have attracted more attention from medical historians than yellow fever (YF). It was one of the most feared of epidemic diseases from the 15th to 19th centuries, when large scale outbreaks in port cities of North and South America, Africa, and Europe caused devastating mortality and helped to shape the expansion of settlements and colonial powers. The landmark studies of Walter Reed in 1900–1901 established that the disease was transmissible among humans via Aedes aegypti mosquitoes [1]. Within one year of Reed's discovery, the disease was successfully controlled in Cuba as a result of vigilant mosquito control campaigns [2]. Twenty-eight years later, yellow fever virus (YFV) became the first mosquito-borne virus to be identified [3]. Despite this legacy, YF is currently classified as a reemerging disease and remains a significant cause of morbidity and mortality, with an estimated 200,000 cases each year and 30,000 deaths [4,5]. Indeed, although a highly effective vaccine is available, epidemiological data suggest an alarming resurgence of virus circulation in West Africa over the last 20 years [6,7]. The failure to implement sustained vaccination programs reflects larger problems of poverty, civil war, and the inaccessibility of rural areas where outbreaks of the disease occur [8].

The agent of the disease, YFV, is a single-stranded, positive-sense RNA virus with a genome of approximately 11 kb. The virus is a member of the genus *Flavivirus* (family Flaviviridae), which contains a number of important vector-borne human pathogens, such as the dengue, Japanese encephalitis, and West Nile viruses. Previous evolutionary studies suggest that YFV originated in Africa, as the deepest phylogenetic split among viral genotypes is between those isolates sampled from East and West Africa [9–11]. What is less clear, however, is the timing and mechanism by which YFV was introduced to the Americas and whether the descendants of the earliest imported viruses still circulate today.

The most commonly cited hypothesis of the origin of YFV in the Americas is that the virus was introduced from Africa, along with *A. aegypti,* in the bilges of sailing vessels during the slave trade. Subsequent to devastating urban outbreaks within port cities on both the east and west coasts of South America, the virus established a sylvatic enzootic cycle within the Amazon, Araguaia, and Orinoco river basins vectored by *Haemagogus* and *Sabethes* mosquitoes [12,13]. Although the hypothesis of a slave trade introduction is often repeated, it has not been subject to rigorous examination using gene sequence data and modern phylogenetic techniques for estimating divergence times [14]. Determining the age of American sylvan YFV is of particular interest given the virtual disappearance of urban (human) YFV transmission in South America in the 20th century. Although a small number of sporadic cases have been reported from residents of urban areas (three cases in 1942 in Sena Madureira, Acra, Brazil [15], 15 cases in 1954 in Trinidad [16], six cases in 1999 in Santa Cruz de la Sierra, Bolivia [17]), there was no evidence for inter-human transmission during these outbreaks. The last documented *Aedes*-vectored epidemic occurred in 1928 in Rio de Janeiro [12]. However, the reinfestation of many densely populated coastal cities with A. aegypti and the emergence of dengue in the Americas indicates that surveillance and monitoring of YF endemic/epidemic viral activity remains a critical public health objective.

To provide an insight on the factors that influence the emergence of YF in the two hemispheres and to determine the time-scale of these events, we have performed an extensive analysis of the evolutionary relationships and dynamics of YFV. To achieve this, we assembled the largest data set of viral isolates compiled to date, including samples taken from a wide range of geographical localities and over an extensive time-span. The fragment analyzed comprised half of premembrane *(prM)* (containing an important cleavage site for pr→M), and extended through the first 112 amino acids of envelope *(E).* This region was chosen because of the relatively large data set available from previous studies [9–11,18–21]. Further, the *prM* and *E* genes are of interest because of their critical role in immunity and infectivity, and because they form the structural proteins of the virion surface and are the primary antigens that induce protective immunity. From these data we were able to infer the time-scale and evolutionary history of YFV, and provide the first direct evidence to our knowledge that YFV was introduced to the Americas during the slave trade.

## Results/Discussion

We examined YFV isolates representing the global diversity of the disease from the World Reference Center for Emerging Viruses and Arboviruses at the University of Texas Medical Branch, Galveston, Texas, United States. Novel sequence data from 37 human isolates, 21 mosquito isolates, and four vertebrate isolates was obtained for the *prM/E* gene region (670 nucleotides [nt], genomic positions 641-1310). Inclusion of an additional 71 *prM/E* sequences from GenBank resulted in an alignment of 133 wild-type isolates representing 22 countries (14 African, eight South American). Table 1 provides summary details on the YFV isolates included in this study (more details are available in Table S1).

**Table 1:**
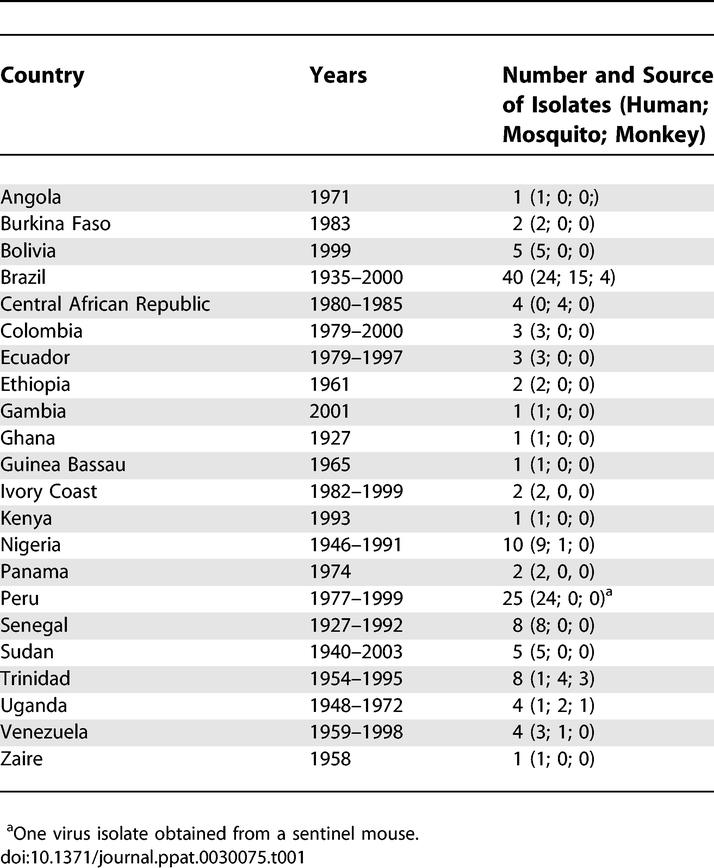
Summary of Yellow Fever Virus *prM/E* Gene Sequences Used in This Study

Our phylogenetic analysis shows that YFV can be divided into two geographic groups, with distinct viral lineages observed in Africa and the Americas (Figure 1). The *prM/E* phylogeny supports the major genotype and subclade distinctions observed previously in phylogenies based on full genome sequences [22], the complete *E* gene [9], and the 3′ non-coding region [23]*.* Four key observations can be drawn from the structure of the YFV phylogeny. (1) The American isolates are monophyletic, (2) the American isolates are divided into those from the east and west of the continent, (3) the isolates from West Africa are most closely related to those from the Americas, and (4) the isolates from East Africa are the most divergent. Such a phylogenetic pattern is compatible with the hypothesis that YFV arose in Africa, most likely in the east of that continent, and was imported into the Americas from West Africa, and then spread westwards across the Americas. To further test this hypothesis, we constructed a model tree in which both the African and American lineages were monophyletic, which is compatible with the theory that the viruses from these two continents have evolved separately for a far longer period of time (an “ancient origin” model). However, the likelihood of this phylogeny was significantly lower (*p* < 0.001) than that of the maximum likelihood and maximum a posteriori (MAP) (“recent origin”) trees, further supporting a more recent migration of YFV from Africa to the Americas.

**Figure 1:**
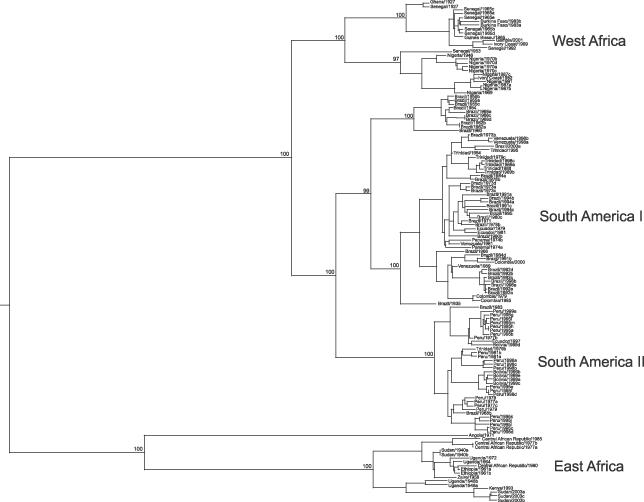
MAP Phylogenetic Tree Based on 133 YFV *prM/E* Gene Sequences The major geographic groupings of YFV are indicated and posterior probability values are shown for key nodes. In all cases, tip times correspond to the dates (year) of virus sampling.

To investigate the timing of the introduction event to the Americas more precisely, we imposed a time-scale on this phylogeny by estimating evolutionary rates and dates of divergence using a Bayesian coalescent approach. Broadly equivalent rates of nucleotide substitution were observed in all data sets studied (countries, genotypes, continents), although the small number of viruses from East Africa precluded an analysis in this case (Table 2). Across all 133 YFV sequences as a whole, the mean substitution rate was 4.2 × 10^−4^ substitutions per site, per year (subs/site/year), ranging from 3.3 × 10^−4^ (South America genotype I) to 8.0 × 10^−4^ (Peru) subs/site/year. These rates are within the range seen in other RNA viruses, including flaviviruses [24]. These rates are also unlikely to have been adversely affected by positive selection on specific lineages or codons. Although the mean ratio of nonsynonymous (d_N_) to synonymous (d_S_) substitutions per site (ratio d_N_/d_S_) was markedly higher in the South American isolates of YFV than in the African isolates, suggesting that selective patterns differ according to local transmission cycle, all d_N_/d_S_ values were less than 0.2, which is indicative of relatively strong purifying selection against nonsynonymous change as commonly observed in arthropod-borne RNA viruses. Further, significant evidence of positive selection (d_N_/d_S_ > 1) was only observed at a single codon, E100, in some data sets, most notably Peru.

**Table 2:**
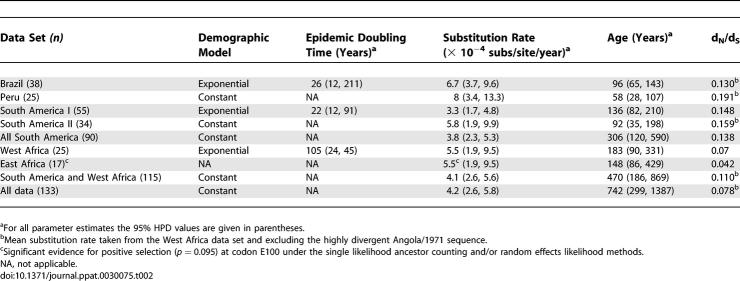
Evolutionary Rates and Divergence Times of Yellow Fever Viruses

From the substitution rates estimated above, the deepest node on the YFV phylogeny, corresponding to the time of origin of viral strains sampled here, has a mean age of 723 years (95% highest probability density [HPD] of 288–1,304 years). A similar time-scale was observed under models of both constant population size (the best-fit to the data in hand) and a Bayesian skyline plot, indicating that these age estimates are robust to the demographic model (unpublished data). More importantly, the estimated mean divergence time of the West African and South American clades was approximately 470 years ago (95% HPD of 186 and 869 years, respectively), while the mean time of origin of both South American genotypes was 306 years ago (95% HPD of 120 and 590 years). Taken together, these analyses suggest that genetic diversity in the available sample of South American YFV arose within the last three to four centuries and provide compelling support for an initial introduction during the period of the slave trade and first contact between the two continents. Furthermore, it appears that the divergence of the two South American lineages occurred shortly after their initial introduction and that they have been maintained in the Americas ever since.

Of equal significance, and no doubt a tribute to effective public health measures, is the lack of evidence for subsequent traffic of viruses between the hemispheres since the advent of the post-vaccine era. An outstanding issue, however, is why the newly established sylvatic South American viruses have failed to cause urban epidemics like those caused by their sylvatic counterparts in West Africa. In West Africa, outbreaks are reported on a nearly annual basis, and five large West African cities have faced YF epidemics since 2001 alone [7]. However, our analyses revealed no major difference in evolutionary dynamics among viruses circulating in South America or Africa, and neither population exhibited the increases in virus diversity expected of epidemic expansion. Indeed, for both regions, the sequence data supported either a constant population size or a population growth rate with an extremely low epidemic doubling time (e.g., ~26 years in the case of YFV from Brazil). This suggests that viral epidemiology in both regions is still dominated by sylvatic transmission.

It is also likely that population subdivision has played a significant role in maintaining the relatively constant levels of genetic diversity within YFV, as reflected in the clear geographic structure of the phylogeny clades, which generally consist of sequences from neighboring countries with little mixing among them. Previous studies of the spatiotemporal distribution of the two South American genotypes [25] indicated that Peruvian YFV may persist in discrete enzootic foci due to biogeographic barriers that inhibit movement of both vectors and hosts. In contrast, YFV isolates obtained during recent Brazilian epizootics exhibit more rapid dispersal over larger distances, suggesting higher rates of virus traffic within the YF endemic zone that can most likely be attributed to human-aided transportation of virus-infected vectors or hosts [26]. Interestingly, these higher rates of virus traffic and population mixing within Brazil appear to correspond to marginally higher rates of population growth (Table 2).

YF provides a powerful historical demonstration of how the establishment of travel and trade routes between countries has been accompanied by the spread of microbes and their vectors. Global trade and transportation in the modern era continues to facilitate the movement of pathogens and vectors farther and faster than ever before, thus altering the potential geographic distribution of infectious diseases. During recent decades there have been several documented cases of the human importation of YFV to non-endemic areas. Since 1964, such episodes have included at least nine documented cases of European and North American tourists who have died as a result of YFV infection after returning home from visits to the Congo, Senegal, Mauritania, Gambia, Côte d'Ivoire, Brazil, and Venezuela [27–30]. These examples demonstrate that although YFV continues to be exported outside of endemic regions, conditions have not been favorable to support secondary transmission. Why YFV has not successfully dispersed to new regions infested with *A. aegypti,* in particular Asia, remains uncertain, although it is clear that previous geographic barriers that prevented spread of YF in the past are quickly eroding.

## Materials and Methods

### Viruses used in this study.

All virus isolates were available from the World Reference Center for Emerging Viruses and Arboviruses at the University of Texas Medical Branch, Galveston, Texas, United States (see Table S1). The majority of isolates were obtained through primary isolation in suckling mouse brain, followed by a single passage in C6/36 cells. A smaller number of isolates had uncertain (undocumented) passage histories, or as many as two to ten passages in suckling mouse brain. The viruses used covered a sampling period of 76 years: 16 isolates from 1927–1959, 19 isolates from 1960–1969, 25 isolates from 1970–1979, 21 isolates from 1980–1989, 46 isolates from 1990–1999, and 6 isolates from 2000–2003. Importantly, all of the South American isolates represented sylvatic transmission cycles, as no YFV isolates representing human-mosquito-human transmission in South America are available in historical or contemporary archives.

### Sequence analysis.

Maximum likelihood phylogenetic trees were inferred for the YFV *prM/E* sequence data (670 nt) set under a variety of nucleotide substitution models in PAUP* [31], including (a) codon-specific substitution rates and (b) the GTR+I+Γ_4_ model, with the rate of each substitution type under the general reversible model (GTR), the proportion of invariant sites (I), and shape parameter of a gamma distribution with four rate categories (Γ_4_) estimated from the data. The GTR+I+Γ_4_ substitution model was also used as the basis to estimate trees using Bayesian Markov chain Monte Carlo approaches implemented in MrBayes [32] and BEAST [33]. The final tree presented is the MAP tree estimated in BEAST (chain length of 25 million, sampling every 1,000), with tip times corresponding to the year of virus sampling.

To test the competing hypotheses of the “recent” and “ancient” origin of YFV in the Americas, we compared the likelihood of the maximum likelihood tree (“recent origin”) with that of a model tree in which both the African and American lineages were monophyletic (“ancient origin”) using a Shimodaira–Hasegawa test [34].

Rates of nucleotide substitution, the age of the most recent common ancestor (MRCA), and demographic histories were estimated for the whole data set and each geographic subset using models that allow for rate variation among lineages under a relaxed (uncorrelated exponential) molecular clock [14] implemented in BEAST [33]. Four population dynamic models were investigated: constant population size, exponential population growth, logistic growth, and expansion growth. To confirm the age of the MRCA of all the YFV sequences analyzed, we also used the piecewise Bayesian skyline plot [35], as this possesses the least constrained coalescent prior. Akaike's information criterion was used to determine the best-fit model, with uncertainty in parameter estimates reflected in the 95% HPD values, and all chains were run for sufficient time to ensure convergence. All estimates were again based on the GTR+I+Γ_4_ model of nucleotide substitution.

Mean and site-specific selection pressures acting on YFV were measured as the ratio of nonsynonymous (d_N_) to synonymous substitutions (d_S_) per site estimated using the single likelihood ancestor counting (all sequences) and random effects likelihood (maximum of 50 sequences) methods, both incorporating the GTR model with phylogenetic trees inferred using the neighbor-joining method available at the Datamonkey facility [36].

## Supporting Information

Table S1 Click here for additional data file. Yellow Fever Virus *prM/E* Gene Sequences Used in This Study
(116 KB XLS)

## Abbreviations

d_N_: nonsynonymous substitution
d_S_: synonymous substitution
HPD: highest probability density
MAP: maximum a posteriori
YF: yellow fever
YFV: yellow fever virus
